# A systems approach to the exploration of research activity and relationships within a local authority

**DOI:** 10.1186/s12961-021-00792-0

**Published:** 2021-11-22

**Authors:** Judith F. Fynn, John Jones, Andy Jones

**Affiliations:** 1 Norwich Medical School, University of East Anglia, Norwich, United Kingdom; 2 Directorate of Community & Environment Services and Public Protection, Norfolk County Council, Norwich, United Kingdom; 3 UKCRC Centre for Diet and Activity Research (CEDAR), Cambridge, United Kingdom

**Keywords:** Evidence-based practice, Public health, Research relationships, Partnerships, Systems, Local authority, Participatory action research, Network analysis

## Abstract

**Background:**

Organizations with responsibilities for public health are increasingly required to use evidence-based practice to inform programme delivery, requiring research to generate relevant evidence, and dissemination and use of evidence to inform decisions and practices. Understanding how relationships between organizational structures, systems and processes influence evidence-based practices is critical to improving practice at both an institutional and system level, yet how these relationships should best operate is not well understood. Understanding how to better support research within local authorities, the elected administrative bodies responsible for services including public health at a regional level in the United Kingdom, is a priority for the National Institute for Health Research (NIHR) Public Health Research. This study is based on Norfolk County Council, a local authority in the east of England. We aimed to apply a systems perspective to develop a better understanding of the structures, systems and processes that support a local authority in becoming research-active, identifying gaps in understanding and recommendations for action to address them.

**Methods:**

Taking a participatory action research approach, we applied qualitative methods to explore research activity and relationships in Norfolk County Council. We surveyed employees and used network analysis to map individuals, departments and external partners involved in research activities and the connections between them. We then applied participatory approaches to conduct a series of focus groups and semi-structured interviews to explore stakeholders’ experiences and perceptions of being involved in research at, or with, the authority, and their ideas for recommendations for future actions.

**Results:**

A range of research activity is undertaken at the local authority, with an emphasis on applied work to improve service delivery. We identified several examples of effective practice and models of research collaboration in some departments. Challenges such as limitations in resources, capacity and knowledge exchange were evident, yet there was a readiness amongst key stakeholders to develop and implement actions that may better support the authority in becoming more research-active.

**Conclusion:**

In large complex organizations, a key challenge is how to share learning across teams and implement good practice at an organizational and system level. Our findings highlight the potential for developing improved collaborative partnership models and systems to support sustainable processes and practices for research and knowledge exchange at an institutional and interorganizational level. The insights gained and shared will support other local authorities and similar large, multilevel organizations with responsibilities for evidence-based public health to explore their own setting and implement change where needed, and provide stimulus for further research into system-level change.

**Supplementary Information:**

The online version contains supplementary material available at 10.1186/s12961-021-00792-0.

## Background

Public organizations with responsibilities for the health of the population they serve are increasingly required to use evidence-based practice to ensure that policy and practice are based on sound evidence. Evidence-based practice requires (i) the generation of relevant evidence, (ii) dissemination to communicate knowledge and information, and (iii) the use of evidence to inform decisions and practices [1, 2]. These processes are critical to ensuring that resources are focused on actions and interventions that have a good prospect of being effective [3]. Failure to do so risks valuable resources being spent on ineffective interventions and/or reduced resources for interventions proven to be effective, and limits the ability of organizations and the wider system to meet public health objectives and targets. Nevertheless, stakeholders with responsibilities for decision-making, and for delivery and evaluation of services and interventions, face several challenges in implementing evidence-based practices [4–7]. Stakeholders involved can include researchers, policy-makers and practitioners from a range of public, private and third-sector organizations. Examples of the challenges in applying evidence-based practice include conducting research that will generate evidence that is relevant to current practice and to future strategies and funding; reporting in a time frame, style and language that is appropriate for a range of stakeholders to make use of the evidence; generating evidence from practice-based projects that is robust to facilitate knowledge mobilization and implementation of good practice; limited stakeholder awareness of alternative approaches to evidence production and use; and generating and using evidence with limited financial resources and methodological skills [7–10].

There has been a growing understanding and appreciation of how factors such as resources, individual and organizational capacity, and organizational structures and systems can act as barriers to or facilitators of research- and evidence-based practice [8–11]. The relationship between the extent to which good practices are embedded within organizations and the development of a “culture of evaluation” or “research culture” has also been discussed within the literature [7, 9]. Schwarzman et al. [9] describe an organizational culture that places value on evaluation and research as a facilitator for staff to take up and use evaluation, and for supporting systems and structures to be embedded within the organization. Previous studies have shown that research–practice partnerships can improve practice, help build individual and organizational capacity to undertake research, and facilitate the development of a research culture within organizational teams [9]. Others have described improvements in adoption of evidence-based practices through such partnerships [12]. However, the degree to which collaborative research practices are embedded within organizations and the nature of relationships can influence the effectiveness of research partnerships and activities [8]. There is a pressing need to improve understanding and implementation of organizational structures, systems and processes that can facilitate initiation and maintenance of research partnerships and networks within organizations and multi-agency systems that have an interest in applying evidence-based practices [9, 13, 14].

In England, local authorities are the elected municipal bodies with responsibility for the delivery of essential public services; these are organized by county and district council, as well as unitary authorities which typically encompass large urban localities, that serve specific geographical areas. Since 2013, local authorities have been responsible for maintaining and improving the health of the population they serve. Some of the benefits of embedding public health within local authorities highlighted at the time public health was incorporated into the local authority remit were the opportunities to work across directorates and departments to address local needs and wider determinants of health [15, 16]. However, such cross-directorate working can be challenging. In the United Kingdom, the National Institute for Health Research (NIHR) was set up in 2006 to “provide a comprehensive research system focused on the needs of patients and the public” [17]. In 2020, the NIHR funded 14 research projects as part of a programme to help them understand how to build a research system that could better support research activities and build research capacity in local authorities [18]. Each of the funded projects within the NIHR Local Authority Research Systems Call were linked to a different local authority in England. This manuscript reports on the findings from one of those research projects undertaken with the Norfolk County Council (NCC) in England.

NCC (hereafter referred to as the Council or the local authority) was used as a case study to explore stakeholders’ experiences of undertaking research activities and collaborating with research partners within a local authority context. NCC serves a predominantly rural county in the East of England with a population of 903,000 in 2019, and a population density of 169 persons per km^2^, making it one of the most rural counties in England. Services are organized within six core departments: Community and Environmental Services (which includes Public Health), Adult Social Services, Children’s Services, Finance and Commercial Services, a Governance Department, and a Strategy and Transformation Department [19].

Over the last decade, NCC has collaborated with research partners, including the local university (the University of East Anglia [UEA]), to jointly deliver and evaluate many projects. Through these projects the Council has increased its understanding of research and its awareness of challenges in evidence generation and dissemination that a local authority might face. Questions have arisen within the Council around the extent to which examples of good practice in research are localized within individual relationships or departments, or are institutionalized and shared across departments and local authorities. This was adopted as a case study theme to explore the relationships between intra- and interorganizational structures and processes, and internal and external influences on research activities and evidence-based practices; developing a better understanding of these is critical to improving practice at both an institutional and system level [9, 13, 14].

Through the lens of a systems approach that would enable us to view the Council and the wider system in which it operates, we explored current research activity, existing research relationships and stakeholders’ experiences of being involved in research activities at, or in partnership with, NCC. For the purposes of this work, research was defined as the systematic inquiry for the generation of knowledge and understanding, and included applied research which seeks to find solutions to everyday problems, and evaluation. Research activities were defined as activities inclusive of conducting research and using evidence from research.

Firstly, we aimed to develop a better understanding of the organizational structures, processes and practices that support a local authority in becoming research-active. Secondly, we aimed to apply the insights gained to understand how lessons from individual projects may be implemented at an organizational level, and what actions may be needed to address gaps within the local network and to support and embed good research practice across the organization. Although the focus in this case study is on a specific local authority, the learning from the research is intended to be applicable to other local authorities and multilevel organizations facing similar challenges, and more broadly those with an interest in or responsibility for systems and practices to support evidence-based public health. To address these aims we identified the following objectives:

### Research objectives

1. To identify existing partnerships, departments, groups and individuals that play a role in, or oversight of, research activity and evidence-based decision-making within the local authority.
2. To explore processes and practices operating within the current organizational structures and systems within the local authority that facilitate research activities, knowledge mobilization and use of research evidence.
3. To identify gaps in current processes and practices in terms of supporting research activities within the local authority, and identify what may be needed to address these gaps.
4. To use these insights to develop recommendations for action to address the gaps, build on strengths and identify how lessons from individual projects and partnerships may be implemented and embedded at an institutional or system-wide level.

## Methods

### Study design

**Fig. 1:**
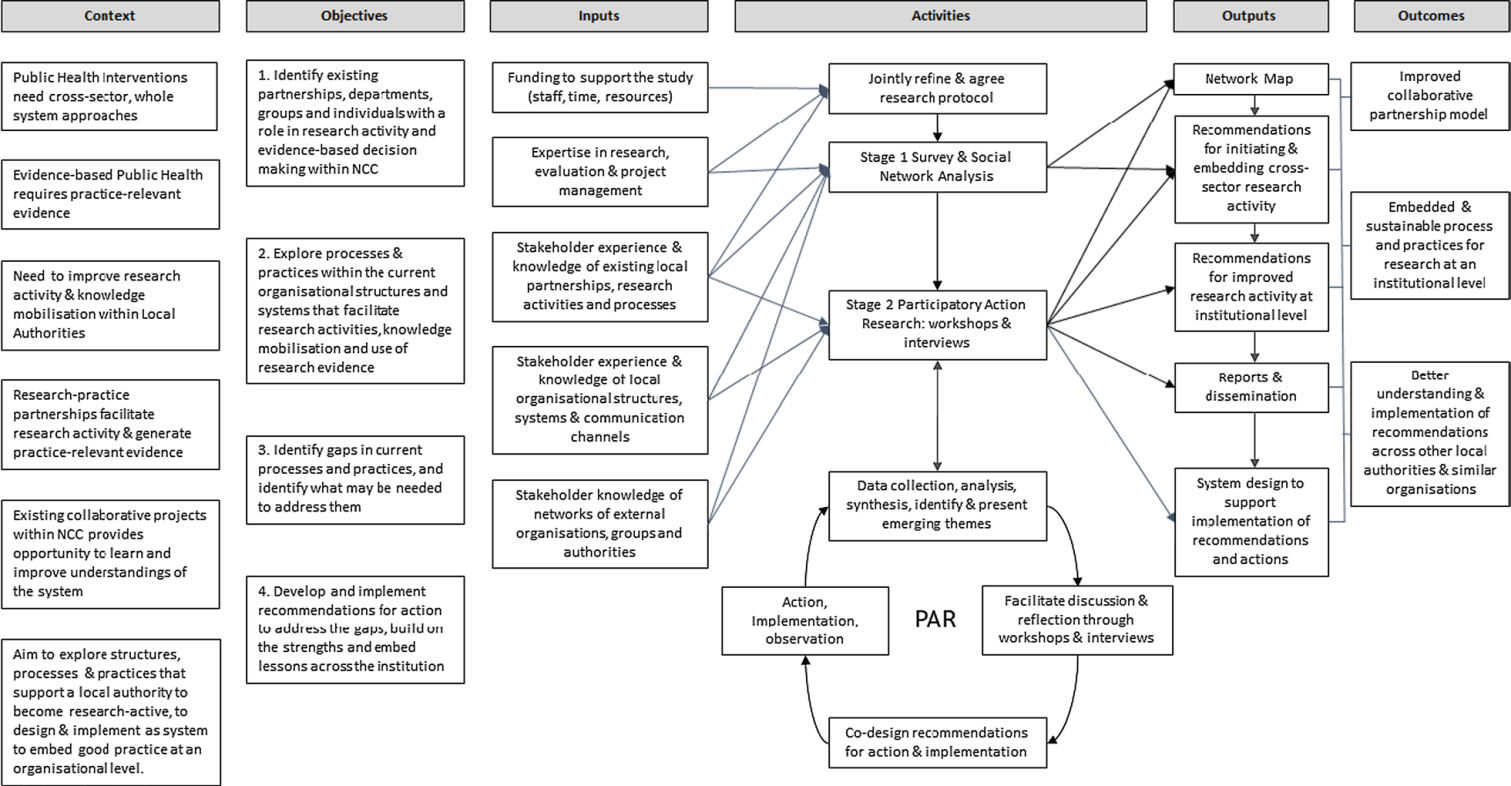
Logic model for the study

The research was a collaboration between NCC and UEA. To address objectives 1 and 2 and explore the processes, practices and factors influencing research activities and relationships within a multisectoral public health setting, we applied a multidisciplinary approach [20]. This was informed by a recognition of the need for a breadth of enquiry beyond the strict boundaries of the local authority, and the boundaries of internal departments and teams, so as to situate the study in the wider system in which the local authority operates and research activities take place. This context is depicted in the logic model we developed to guide the research (Fig. 1).

The research was conducted by applying qualitative methods across two stages. Firstly, we applied network analysis [21] to understand how the local authority and partner organizations may be viewed as a system in which research activity sits. Network analysis is a way of mapping and developing a visual representation of the key players (often termed “actors”) and relationships. It is a method that can be used as a descriptive and diagnostic tool [22]. Secondly, we applied participatory action research approaches that involve the input of those key players [23] to allow us to engage and work collaboratively with stakeholders from the local authority and related organizations, to adapt our methodologies in response to emerging stakeholder requirements and priorities, and to collaboratively seek recommendations for action.

### Stage 1: Network analysis

#### Data collection for the online survey

We used an online survey to identify individuals in the local authority that are engaged, or have an interest, in research activities as part of their work. To explore the breadth of research activities and how they may be used, it was important to ensure stakeholders had a shared understanding of what we meant by the term “research activity”. As defined in the background, research activities were defined as inclusive of conducting research and using evidence from research.

To ensure we reached as many staff as possible across all departments and teams at the Council, we contacted the directors of all departments and heads of service teams, as well as the internal communication team, to provide them with the details and link for the online survey and to ask them to share this with all staff. The survey remained open for the duration of the study (4 months), although no responses were received after the second month.

The survey was designed and agreed upon by all authors, and asked respondents 15 questions about their involvement, or interest, in undertaking or using research as part of their work in the local authority. This included asking them to identify up to 10 people that they currently collaborated with or had collaborated with in the past 2 years for research purposes, and to state whether those partners were employed within the local authority or were from an external organization. We included two categorical questions to help understand the nature of the relationship and communication with each identified partner. Firstly, respondents were asked to select the most appropriate description of the communication: formal (e.g. scheduled meetings), ad hoc as required (e.g. to ask a specific question or respond to a specific question), mixture of ad hoc and formal, or by chance (e.g. only when your paths cross). Secondly, they were asked to select the most appropriate description of the frequency of contact: rarely (e.g., We hardly ever communicate unless we need a specific piece of information or other input), occasionally (e.g., There may be long periods when we are not in contact during a project, but we will be in contact at key milestones), frequently (e.g., We are in regular contact throughout our collaboration), or very frequently (e.g., We are in contact at least weekly when we are working together; we always know what is happening in relation to each other’s work).

#### Data analysis for the online survey

After the survey had been available to participants for 2 months, the survey outputs were exported into a Microsoft Excel file for cleaning and data management. Each respondent and named partner were given a unique code to de-identify them. Each person was also coded with attributes based on the survey responses, including whether they were a respondent or named partner; their organization, team or department; and their engagement with or interest in research activities. The coded data were then imported into the UCINet software package [24], where they were used to generate network maps to describe the connections between stakeholders, internal departments and external research partners.

### Stage 2: Focus groups and semi-structured interviews

The second stage of the research was conducted over three phases of data collection, each with a different purpose (as shown in Table 1). In line with a participatory action research approach adopted, the research was iterative, and the themes and findings identified in each phase were used to inform the subsequent phase. In this way, the focus groups and interviews were used to provide feedback on the findings from the preceding phase, and to facilitate discussion around emerging issues and themes to gain a fuller understanding of stakeholders’ experiences and perspectives (Additional file 1 provides details of the supporting material provided and semi-structured questions). To allow this circular action research approach, the focus groups and interviews for each of the three phases in stage 2 were conducted sequentially over the final 3 months of the study.

**Table 1:** Description of each phase of data collection within stage 2 of the study

Phase	Purpose	Participants (total number)
1	To explore internal stakeholders’ experiences of research relationships and the types of research activities undertaken	Three focus groups (*n* = 10) Four interviews (*n* = 4)
	To explore external stakeholders’ experiences of research relationships and the types of research activities undertaken	Two focus group (*n* = 7) Four interviews (*n* = 4)
2	To collaboratively develop case studies to explore approaches adopted within internal teams to facilitate research activities and partnerships	Three focus groups (*n* = 9) Five interviews (*n* = 5)
3	To explore preliminary findings and provide opportunities to feed into the study conclusions	Two focus groups (*n* = 12) Presentation and discussion with the local authority corporate board

#### Study sample

Purposive and snowball sampling approaches were applied to identify potential participants to include in the second stage of the research. Initially, survey responses were used. All respondents who indicated their willingness to participate, and who had shared their email address with us via the survey, were contacted and invited to participate in a focus group or interview. We also used survey responses to identify named external partners; where these people had their contact details readily available on organization websites, we contacted them to provide details of the study and to invite them to participate. In addition, employees who had key roles related to research activities at the Council, such as staff involved in data analytics, research governance or working in research-active teams, were contacted and invited to participate in phase 1.

In phase 2, using the findings generated from phase 1, we identified six examples of different approaches to research activities being undertaken by different teams that involved staff located within Community and Environmental Services, Adult Social Services, Children’s Services, and the Strategy and Transformation Department. We contacted key informants from each of these groups to invite them to participate in an interview or focus group to develop a case study that could be used to (i) showcase their research approaches and practices, (ii) share examples of good practice and (iii) help identify approaches to facilitating research and challenges they face in engaging in research, that may help inform future practice and support research capacity-building within other departments or teams. Stakeholders from four different departments responded and collaborated to develop four case studies.

In the third phase, we sent an invitation to all stakeholders who had participated in any of the interviews or focus groups to participate in a focus group to discuss the findings of the study and to provide the opportunity to comment and feed into conclusions and recommendations. In this final phase of the research, findings were also presented to the corporate board (governing body) of the Council for comment.

#### Data collection for the interviews and focus groups

Supporting material and a topic guide with indicative questions were developed for each of the three data collection phases in stage 2 of the research (these are provided in Additional file 1). These were sent to participants to facilitate reflection on their experiences and practices in advance of each focus group and interview, along with a participant information sheet and consent form to be signed prior to further participation in the study. In phase 1, eight questions were included that focused on exploring the types of research activity that stakeholders were engaged in, and their experiences of research activity and research partnerships. In phase 2, seven questions focused on how research practices had evolved in specific teams, the benefits and challenges of the approaches and practices they adopted, and stakeholders perceptions on how these approaches may fit across other departments and teams within the local authority. In phase 3, initial findings from the previous research phases, including the network map, were used as prompts for discussion to explore potential next steps for promoting and supporting research activities across the local authority.

Focus groups lasted approximately 60 minutes and had between three and four participants in each, whilst interviews lasted between 26 and 50 minutes. Focus groups were facilitated by JF and/or AJ; all interviews were conducted by JF. Focus groups and interviews were conducted using Microsoft Teams and recorded on an audio-recording device. These were then transcribed by JF.

#### Data analysis for the interviews and focus groups

An inductive approach was applied to identify key themes in the transcribed data following phase 1. These initial themes were used to develop a coding framework, which was discussed and agreed to by all authors. This was then applied to code the data generated from each of the phases of stage 2, with additional emergent codes added iteratively. In addition, a set of case studies were developed as examples of research approaches adopted within teams at the local authority.

## Results

The findings are presented as a narrative synthesis, linked to the stages of the research.

### Stage 1: Survey and network analysis

After removal of eight incomplete responses, the survey sample consisted of 104 participants. Of these, 54 (52%) stated that they were either currently engaged in doing research or had been in the last 2 years, and a further 43 (41%) respondents stated that they were not engaged in research but were interested in doing so. Some 68 (65%) were currently engaged in using research evidence or had been in the last 2 years. Respondents identified 174 partners that they collaborated with for the purposes of research; this included 69 internal partners that had not completed the survey and 105 external partners.

Respondents described the nature of collaborations and communication with partners variably. In total, 217 relationships were identified. The most common categorization used to describe the nature of communication was “a mixture of ad hoc and formal” (*n* = 118, 54%), followed by “ad hoc” (*n* = 54, 25%), “formal” (*n* = 41, 19%) and then only 2% (*n* = 4) describing communication as “by chance”. Frequency of contact within relationships was generally high, with these described as “very frequent” in 27 (14%), “frequent” in 79 (42%), “occasional” in 59 (31%) and as “rare” in only 23 (12%) of relationships.

#### The network of research relationships

**Fig. 2:**
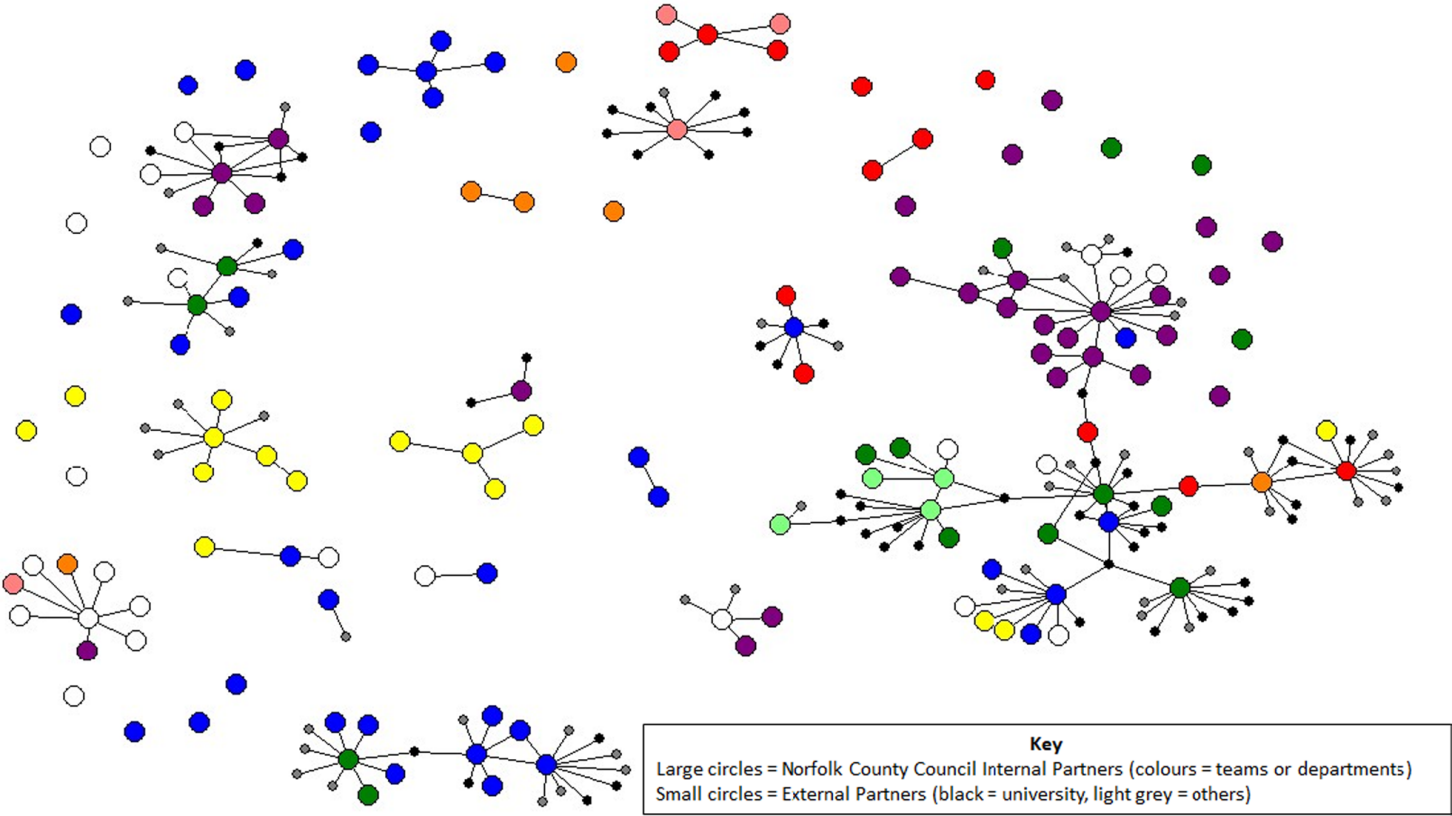
Network map to show individuals engaged in or with an interest in doing or using research, and the partnerships they identified

Figure 2 shows the network map of individuals and their connections to internal and external partners. Internal partners are colour-coded by department or team (e.g. Public Health, Insight and Analytics). To preserve anonymity, these teams are not labelled. External partners are coded as “university” or “other”.

The map shows several relationships between the local authority and university partners, primarily the local university, but also other universities in England and across Europe where there are connections through specific research projects. The category grouped as “other” includes research partnerships that were less frequently mentioned, such as other local authorities, government departments, quasi-governmental organizations, research networks, professional associations, the public, and charitable and voluntary organizations.

The map also shows that stakeholders from a wide range of departments are involved in research activities. It also shows clusters of research relationships, with several clusters around individuals who connect groups and may act as important links within the network. The map also shows several examples of interdepartmental research collaborations, along with isolated stakeholders who have not described themselves as connected to others through research.

### Stage 2: Focus groups and interviews

#### Phase 1: What are the types of research activity that stakeholders are engaged in, and what are their experiences and perceptions of research activity?

Stakeholders described various examples of research activities. These included ongoing use of evidence in service improvement and development plans; public consultations; drawing on evidence from other local authorities informally and formally; and devising tools, methods and interventions, testing implementation, and evaluation. Some stakeholders thought there were differences in how people across the local authority would interpret research; for example, things like quality assurance and evaluation may be considered “business as usual” and not categorized as research if they do not have wider applicability.

Stakeholders emphasized the importance of research being applied, and outputs needing to focus on service development and improvement for the people across the County. One stakeholder commented:

*We are very evidence-based, and feel we shouldn’t be making decisions unless it is evidence-based … It is public money, so we should be squeezing every drop of value out of it, and for me that is what research is about, to understand things and to make things better. We need to use research to inform the things we do.*

The benefits of bringing grant funds for projects and their value in enabling assemblages of tailored teams to address specific issues, “out-of-the box” thinking, and proof-of-concept testing before embedding systematic change were all highlighted. Participants also acknowledged that project work is time-limited, and once a project is completed, the knowledge gained is not always retained. It was felt that within departments and project teams there are people with transferrable research skills that could be used across the service and in other departments with wider sharing, and that there are missed opportunities for learning and knowledge from the practices of research to be shared across the Council. As one participant commented:

*Working at the local authority has been a great experience for me, and it has given me time to do research, but maybe fewer opportunities to say what we have done. I think we need to celebrate it a bit more*.

We identified several key themes related to participants’ experiences of research activities and research relationships, as shown in Table 2. These themes show important factors that stakeholders described as challenges or facilitators to being research-active within their role at the local authority.

**Table 2:** Themes related to stakeholders’ experiences of research activities

Key themes	Examples of challenges and facilitators
Research activities	
Limited awareness and knowledge of what others are doing	Challenges are associated with being a large organization that fulfils many functions Duplication of efforts and missed opportunities for greater efficiency Fluidity of roles across different departments Communication is important to help people know what questions to ask, how to find answers and who to ask
Limitations in resources	Limited financial, analytical and time resources No specific people managing research Lack of resilience and fragile staff teams
Alignment of research with long-term strategy	Importance of applied research that will develop and improve service is recognized Challenges of knowing how outputs will be used Limitations in the capacity to align research to longer-term strategic needs Longitudinal studies are difficult within an applied context, and traditionally not done The balance between time spent now for better working in the future needs to be improved
Research relationships	
Openness to collaborating with external partners	Range of projects with internal and external partners Good relations with universities, particularly local ones and those with relevant expertise Existing and new networks, e.g. health and care partnerships, data analytic networks, local practice networks Partnering with external companies and consultants is a newer way of working and needs developing Challenges in working with dispersed groups and timelines for feedback Benefits of access to research expertise, tools, external funds and improved capacity to do research
Collaboration, networks and knowledge-sharing	Based on relationships built over time, informal, personal connections New links remain based on existing relationships where there is trust Networks may not be accessible to all staff (e.g. mainly limited to directors of teams) Balance between naturally forming relationships and putting a structure on that (potential resistance) Trade-offs between collaborative approaches and time spent learning on the job doesn’t always favour networks of learning
Suggested developments	Development of a knowledge hub Engagement of staff with responsibility for liaison and facilitating research Framework for collaborations and capacity-building, training element, working across departments and opening minds Moving from informal connections to systemized and enduring partnerships

#### Phase 2: Case studies as examples of research activities

We identified several examples of collaborative research, internal and external research partnerships, innovative approaches and good practice across the local authority. We collaborated with stakeholders to develop four case studies as examples of differing approaches and models of research activity within different local authority teams or departments (these are provided in Additional file 2). Table 3 provides a summary of the different approaches to research identified in the case studies, and the key strengths and challenges that stakeholders described as being associated with these approaches.

**Table 3:** Approaches to research identified by stakeholders involved in the case study development

Approaches to facilitate research activities within local authority teams/departments	Strengths and challenges associated with these approaches described
Project-based research–practice partnerships between the Council and universities	Brings access to academic expertise and advice Exposure to new ways of working that support skills development and capacity-building Brings credibility that can improve buy-in from internal and external stakeholders Can bring in external funding Good communication and relationships are needed Short-term nature of projects can be a challenge to long-term planning
Leveraging existing connections to establish working relationships and interagency partnership in response to shared needs or concerns (e.g. response to COVID-19)	Mutually beneficial research collaboration in which all partners, services and wider stakeholders gain Established connections are key to initiating new collaborative projects rapidly Engagement in collaborative work strengthens relationships and increases opportunities for ongoing or future collaborations
Evolving models of collaborative working (e.g. joint funding of research, commissioning research, providing data, interventions or participants for external research, collaborative/co-developed research)	Shifting model as relationships are built and embedded Shifting model as individual and organizational capacity to engage in research is built and embedded Differing models allow flexibility and adaptation to the needs of specific projects
Departments where research culture is established and embedded and/or staff and teams are research-ready or research-active	Provides a level of autonomy that allows flexibility to take opportunities Challenges include being restricted by time scales, budgets and other work commitments Relies on proactive efforts of staff in looking for opportunities to do research, bring in external funding and develop partnerships Brings skill sets for research Brings connections for research
Engagement between departments, including formal and informal arrangements for fixed shared posts or resource across departments	Helps build relationships Improves sharing of insights, learning and resources Improves internal network Builds capacity and skills Builds confidence around joint working
Dedicated research staff within departments or the organization	Central support to facilitate research, training and capacity-building Develops and embeds a culture of valuing and using insight and evidence for research Central role helps to understand and align research with longer-term strategies Ensure research and collaborations are practical and meaningful to the Council and stakeholders
Collaboration platform	Having agreed platform facilitates processes in setting up collaborations and auditing, and overcomes some of the challenges in setting up contractual arrangements and procurement

#### Phase 3: Key themes identified from the final workshops and next steps

Stakeholders thought the study had been a good starting point to bring people from different teams and departments together, and to start conversations about what more could be done. The mapping was seen to have been useful to stimulate discussion around how the networks may be developed and shaped going forward. Bringing people together in the focus groups and showcasing research activity through the case studies was thought to have helped develop a better understanding of the breadth of ongoing research activity and opportunities for future collaboration. Stakeholders expressed a desire to engage in further discussion around how best to build on the study and its findings, and to develop and implement interventions that may better support the authority in becoming more research-active. Table 4 shows the key themes identified by stakeholders as important for informing potential recommendations and implementation.

**Table 4:** Themes identified by stakeholders as important for informing recommendations and implementation

Themes	Factors	Potential next steps
Build on existing strengths, resources and good practice	Capitalize on (i) new and ongoing collaborations, (ii) existing collaboration platform, (iii) recent COVID-19 work that has helped unlock benefits of sharing knowledge and skills across organizations	Explore ways to share skills, resources and good practice Link stakeholders internally Move from ad hoc to more systematic and embedded relationships and research arrangements Celebrate and share successes
Training and building capacity for research	Focus on (i) working across departments and with universities; (ii) using and extending existing models currently operating within some departments	Identifying and implementing a range of training models, e.g. secondments, apprenticeships, champions, internships, professional development programmes Engaging staff with responsibilities for promoting and facilitating research and partnerships
Strengthening networks across departments and with external partners	(i) Balancing Council needs for knowledge that cannot be met internally with what works for a university, educationally, professionally and financially (ii) Moving from informal connections and isolated projects to systemized and enduring relationships and activities (iii) Increasing requirements for universities to show impact offers opportunities for applied research	Build relationships and identify mutual benefits Develop a framework to facilitate research, collaboration and capacity-building Develop a knowledge hub to facilitate sharing or knowledge and resources
Alignment of research activities with the strategic short-, medium- and longer-term needs	(i) Interest in exploring key issues the County faces, and potential for innovative projects and joined-up thinking that could draw on non-typical resources to find interventions to address these needs (ii) Coproduction is increasingly valued and required	Identify a handful of projects that can be used to help formulate a structured approach to identify short-, medium- and long-term research priorities for the Council

In thinking about potential next steps, stakeholders highlighted the importance of recognizing the nature of funding within the public sector and resource limitations, as these concerns will continue to mean that research activities will typically need to be shaped around short-term project work. Capitalizing on existing strengths and capacity within the organization and recognizing the added value of project work and partnerships were seen as key to enabling change. There was also interest in thinking about the issues the County is going to be facing in the recovery period following the COVID-19 pandemic, such as the economic situation, mental health concerns and long-term health issues such as post-COVID syndrome (otherwise known as long COVID) [25]. Stakeholders thought that this brought potential for innovative projects and joined-up thinking that could draw on non-typical resources to find interventions to address these needs; one example given was to look at the potential role for library and museum services to improve health and well-being.

## Discussion

This study found strong evidence of embedded good practice in relation to conducting research and using associated evidence to inform service delivery in some teams, and strong collaborations within sections of the local authority. There was a clear focus of interest amongst stakeholders across the authority on research that is applicable and that will improve the service and outcomes for the people it serves. The value of research projects to access funding, and to allow innovative thinking and testing before embedding systematic change, were recognized. Yet stakeholders also emphasized challenges, such as limitations in alignment of research activities with longer-term strategic needs, limitations in resources and capacity for research in some teams, and a lack of awareness of what research activities other teams were doing. Stakeholders highlighted missed opportunities for shared learning, shared resourcing and knowledge exchange, and for service improvements and efficiencies that this would allow.

Many of the challenges identified in this study are typical of large multisectoral and resource-limited organizations, and of siloed working. For example, there was strong evidence of research being conducted within many departments, yet this was generally carried out by individuals or groups within discrete projects, often with fixed duration and funding. These findings align with those of previous studies that have explored the functioning and challenges of public health services within local authorities [16, 26], and of implementing evidence-based practices in public health or real-world settings [9, 10]. From a local authority perspective, it is critical to understand the benefits of research, how it can be used to improve services and productivity and to provide public benefits. It is important to explore and consider how the organization may best invest in research, how return on investment is measured, and how research could inform a framework for short-, medium-, and long-term goals. Resources, including staff, time, funding and analytical resources, were identified as critical to enabling research activities and to facilitating capacity-building and development of a research-active workforce. Resources and a research culture were also thought to be essential to allow the initiation, development and sustainability of research relationships and networks, which in turn supported the embedding of a research culture and good practice within teams.

The findings also support previous studies that have highlighted the benefits of research–practice relationships, and the importance of understanding how those relationships can influence practice [8, 9, 27, 28]. Such benefits include building individual and departmental capacity, and providing access to tools, expertise and external funds to do research. The importance of existing relationships in developing new relationships, providing opportunities for collaborative projects, and building capacity and embedding a research culture was highlighted by many stakeholders. Leveraging existing relationships and making better use of stakeholders with transferable research skills were thought to be important strategies to improve knowledge exchange and address some of the challenges and missed opportunities for greater efficiencies and capacity-building. Findings from the case studies illustrated that where there were existing relationships, these were more easily called upon when needed. One such case was the partnership working in response to the COVID-19 pandemic that enabled working relationships to be initiated rapidly and effective working practices to be established to facilitate sharing of data and relevant evidence across service teams and organizations.

Recognizing the value of leveraging existing relationships, within the context of this study, network mapping was a useful tool to identify key stakeholders that could connect others, and individuals and groups that appeared to operate in silos that may benefit from greater connectivity. Thus the value of network mapping was not just as a descriptive or diagnostic tool [22], but as a tool to prompt discussion and stimulate solution-seeking activities about how to leverage existing connections and to better connect individuals and teams internally and externally. Its use was critical to understanding the wider system in which research activity within the local authority sits, and to applying a participatory action research approach that could respond to emerging findings and stakeholder priorities to generate data that could inform actions and change [23].

The collaborative and iterative methodology applied enabled us to identify key themes, and also revealed a range of different collaboration models operating within different teams. The findings showed evidence of evolving working practices, with a shift towards a greater focus on internally led research and coproduction as research relationships, capacity and cultures became embedded. Thus, the collaborative models can be viewed as a continuum; for example, moving from engagement of external research partners in a consultative relationship or providing access to data, services or participants for externally led research at one end, to co-produced jointly led or internally led research projects and research expertise embedded in the staffing structure at the other. Stakeholders within research-active teams recognized that a flexible approach to adopting different models allowed adaptation to the needs and nuances of specific projects, research and opportunities. Having stakeholders and research expertise embedded within the organization may be critical to the organization’s ability to recognize the value of differing approaches and to capitalize on opportunities for research, collaboration and funding. The findings highlight the importance of understanding and implementing organizational and staffing structures and systems that can facilitate processes and practices to support research- and evidence-based practices, as discussed elsewhere [8, 9]. Further, the study highlights the importance of understanding the wider system and opportunities for mutually beneficial interdepartmental and interorganizational relationships.

This work suggests that there remain several key questions to be answered: What model is appropriate in organizations, such as local authorities, to support collaborative research? How do such organizations, and individual staff, get more involved in research activities? How can lessons from discrete projects be shared to improve practice at the organizational level? And how can organizations ensure that research activities are used to drive decisions that facilitate continuous service improvement, and are effective and transparent?

### Strengths and limitations

The strengths of this study include the collaborative approach and the use of systems approaches, such as network mapping, to facilitate this. Prior to the commencement of the project, the first author was a university researcher independent from the Council. They were however employed by the Council for the duration of this research study, although they operated in an independent manner. Having the researcher embedded in the Council for the duration of the study facilitated access to people within the organization and allowed trust to be built and multiple perspectives to be gathered. Collaborating with key stakeholders using our methodological approach allowed us to capture data from a wide range of departments and activities to provide an overview of the diversity of research practices and experiences. An additional key strength of the study was the timely and broad dissemination; findings were fed back to staff and heads of departments at the Council and to the elected governing board, and have also been reported to the Department of Health and Social Care (the government body responsible for public health in the United Kingdom).

There were limitations in our ability to rapidly reach the target population for the survey. This was influenced by the short time frame for the study (4 months), the context (the 2020–2021 COVID-19 pandemic), and the complexity of the organization and its communication channels. Survey responses therefore represent a select sample of individuals from a very large and complex organization, and the results likely underrepresent the full extent of research activities taking place and stakeholders engaged. It should also be noted that departments are likely to be differentially represented; for example, it is likely that the most research-active individuals responded, and those in departments at the heart of the response to COVID-19, such as Public Health, are underrepresented. The findings should therefore be viewed as a sample of the population only, and as a snapshot at a given time. Nevertheless, the map serves as a starting point for discussions around how the network may be shaped to capitalize on existing research relationships and resources, and further developed to facilitate knowledge exchange and capacity-building to conduct and use research.

## Conclusion

There are clear benefits for local authorities and similar organizations from initiating and embedding research–practice partnerships and collaborative working models, conducting applied research, and making use of evidence to inform service delivery. In large complex organizations, which are often resource-limited, a key challenge is how to share learning across teams, and to move away from siloed working and implement good practice at an organizational level. Better understanding of how project work can influence organizational policy and governance and how a collaborative platform could be further improved to deliver long-lasting and sustainable improvements is needed to bring about action and effect change. It is crucial that any system or actions proposed for implementation are cost-effective, realistic and achievable.

In adopting a collaborative participatory action research approach for this study, its impact is centred around the potential for outputs to be translated into actions that are implementable and that bring about changes in practices, processes and systems, as illustrated in the logic model for the case study organization (NCC) (Fig. 1). The anticipated impact in the short term will be evidence of an improved collaborative partnership model and a system initiated and embedded to support sustainable processes and practices for research and knowledge exchange at an institutional level. In the longer term, the insights gained are intended to be applicable to any organization seeking to develop research- and evidence-based practices, and will be of particular value in supporting other local authorities and similar large, multilevel organizations to explore their own setting and implement recommendations where applicable. There would be value in further research to evaluate implementation of actions taken in respect of the findings from this study, and their impacts on organizational or system-wide changes and capacity for research.

## Supplementary Information

**Additional file 1.** Themes and questions for Interviews and Focus Groups.

**Additional file 2.** Case studies of differing approaches and models of research activity within different local authority teams or departments.

## Data Availability

Data sets used and analysed during the study are not publicly available because they contain information that could compromise research participant consent and anonymity. Data sets are available from the corresponding author on reasonable request, and subject to permission from NCC.

## Abbreviations

NCC: Norfolk County Council
UEA: University of East Anglia
NIHR: National Institute for Health Research
