# Supplementary material for: A systems approach to the exploration of research activity and relationships within a local authority
Source: Health Res Policy Syst. 2021 Nov 22;19:137. doi: 10.1186/s12961-021-00792-0 (PMC8607228; doi:10.1186/s12961-021-00792-0)
Supplement: Supplementary file 1 — Additional file 1. Themes and questions for Interviews and Focus Groups. [file 12961_2021_792_MOESM1_ESM.docx]

**Themes and questions for Interviews and Focus Groups**

**Phase 1: Supporting Materials: Logic Model, Study Design, Introduction to the study**

1. What do we all understand by “doing or using research activities” at or with a LA?
2. What are the main types of research activities that a local authority does?

I would like you to think about the research relationships you have:

How important are these relationships to NCC being an effective organisation? Why do you feel that?

In your experience what happens to allow those relationships to form? (e.g. ad-hoc meetings, established collaborations, contractual obligations)

What mechanisms are used to share information via these relationships?

What are the key facilitators and barriers to the relationships forming and then working effectively?

1. What do you think local authorities such as NCC could do differently to make better use of research focussed relationships?
2. “What is the biggest issue that needs to be tackled with respect to research participation at local authorities such as NCC”?

**Phase 2: Case Study development**

Thinking about the specific research activities that you are involved in, or your ongoing practice with regards to research activity if that is more appropriate.

1. Please can you briefly describe the research or types of research you do.

prompts: How did this come about? Who is involved (partners, departments etc)? How did you initiate and develop those connections and involvement? Who performs what roles in the research relationships? Is this project based, or an ongoing relationship?

2. What does doing or using research mean to you in the context of your role in the local authority, or how would you describe your research practice and experience?

3. Please can you describe how your research practices have evolved, and how this evolution has been shaped by the constraints of the local authority.

4. What do you feel are the benefits of adopting the approach and practices you have described?

5. What do you feel are the challenges of adopting the approach and practices you have described?

6. What are the things you would like to do differently but can’t?

What are the reasons for that?

7. To what extent do you feel your approaches and practices might they fit across other departments and teams within the local authority? What might the challenges of implementing them more widely be?

Phase 3: Supporting Materials: PowerPoint slides to share initial findings and network maps

1. Does this reflect your views, is this what you had expected, any surprises?

2. What actions are already being taken to address any challenges?

3. What do you feel may be needed to contribute to this, or done differently?

4. What do you feel needs to happen next?
