# Supplementary material for: A systems approach to the exploration of research activity and relationships within a local authority
Source: Health Res Policy Syst. 2021 Nov 22;19:137. doi: 10.1186/s12961-021-00792-0 (PMC8607228; doi:10.1186/s12961-021-00792-0)
Supplement: Supplementary file 2 — Additional file 2. Case studies of differing approaches and models of research activity within different local authority teams or departments. [file 12961_2021_792_MOESM2_ESM.docx]

### Case Study 1: Norfolk Office of Data and Analytics “Reasonable Worst-Case Scenario Planning” – Mutually beneficial joint working

This work is part of the Councils ongoing response to the Covid-19 pandemic. The research group formed in Spring 2020 and brought together analysts from NCC, UEA , and specialists from the Norfolk Hospital Trusts, Social Care, Mortality Group and Districts to model likely numbers of cases of Covid-19, what that would mean for hospital admissions and the number of deaths in the system, and the impact on other care and services. Effective modelling was facilitated by early data sharing by the STP. The group met every week.

In Autumn 2020 the group were asked to support winter planning for NCC, Social Care services, and hospitals in order to understand their required capacity. Working with researchers from UEA to look at the association between the number of expected cases and the number of hospital admissions, the group modelled a “Reasonable Worst-Case Scenario”. Critically, they were able to apply national research to translate into the local scenario.

This example of partnership working came about because stakeholders from the NCC Intelligence and Analytics Teams reached out to Norwich Medical School, leveraging existing connections and established working relationships with UEA. Conversations with existing internal and external partners, including the Insight and Analytics Lead for the ST, and an understanding of the shared concerns was key. A member of the group reflected:

*“A degree of co-incidence, but it was useful, and we have kept it going. Out of it we got the initial wave model, we were able to look at the Covid-19 Protect Model, we were also able to start sharing data and information across the system, so partners could look at what was going on and provide their expertise to understand it better. We were also able to start discussions about the Social Care and infections in Care Homes, it just facilitated a good discussion.”*

The impacts of this work are ongoing; for example, it is being used by SCG, and Adult Social Care to plan their hospital discharges. Members of the group also recognised the importance of mutual benefits in partnership working; and identified the value to research partners of the Council sharing data and information, and their knowledge of systems where you can access information and data. In discussions it was concluded:

*“The work has just strengthened those relationships, and so I think we will continue to work closely with partners across the system. That long-term relationship between the team and the University through professional work, needs to be there all the time. You need to have that relationship up and running and investment in that relationship before you can really draw on it in earnest like we have done.”*

Key strengths of this approach were seen as:

- Mutually beneficial research collaboration in which all partners, services and wider stakeholders gain.
- Established connections key to reaching out and initiating new collaborative projects, and also to being able to do that in a timely manner to respond rapidly to emerging needs.
- Engagement in collaborative work strengthens relationships, and brings additional opportunities for ongoing and further collaborations.

### Case Study 2: Active Norfolk - A journey to embed research within the organisation’s culture and practices

Active Norfolk established good relationships with UEA that enabled them to develop an evolving model of collaborative working. They have been involved in several projects with differing models to support research and evaluation. These include projects where a university researcher was jointly funded through Active Norfolk, interventions where the university has been commissioned to conduct an evaluation, projects where they have been approached to deliver an intervention for university led research, and collaborative and co-developed projects. It was stated:

*“The opportunities are not always the same, but it has demonstrated the ways we can work together.”*

The team recognise benefits of working with academics, such as having external and independent input and credibility, as well as the value of engaging with subject experts in research to grow the organisations understanding of research. A significant focus of the organisation has been building capacity in the wider team to embed a research culture. From those initial research relationships and their understanding of insight and research, their approach evolved into having a dedicated internal Evaluation and Insight Officer. A member of the Active Norfolk management stated:

*“I think the most significant step for us as an organisation was building capacity in our own organisation to fulfil some of that research function; to build our own approach to research and establish approaches to collaborating with academic partners.”*

The approach has evolved further by developing formal and informal arrangements to work with other local authority departments, such as the Environment Team and Public Health. Having fixed shared posts or resource has helped build relationships, and to share insights and learning through meeting with senior level colleagues regularly.

*“It is about having gatekeepers and a willingness to work across departments. Being networked internally within our organisation, with the team, with the senior team, and also more recently within the County Council and the Intelligence Network, it helps people know what is possible so they enhance their skills but also what others are doing.”*

Key strengths of this approach:

• Central support and facilitation for the organisation which helps secure buy-in and a culture of valuing and using insight and evaluation across the team.

• Having that central role integrated within the organisation's leadership team and attending senior management meetings helps to understand the direction of the organisation and the work of other departments, as well as advocating research practices at a senior level.

• Being part of a wider intelligence network within the Council facilitates learning from others, shared resources, making contacts and finding out about tools and training.

• Having someone with research skills inside the organisation to facilitate research relationships and to ensure collaborative work is practical and meaningful for the Council, Active Norfolk and research partners.

### Case Study 3: Norfolk Museums Services – Responding to opportunities to do research

Research is a normal part of the work for staff in the Museum Service to keep themselves informed, but more importantly to underpin programming. Occasionally opportunities arise for specific projects that are linked with contemporary agendas or Council priorities. Two example of projects that illustrate the pro-active approach to respond to research opportunities provided below:

**The Covid -19 Social History Research Project** was a response to the Covid-19 pandemic. Curators from across the service each had their own response appropriate to their setting and audiences: putting out appeals for information, surveys, Facebook campaigns, and asking participants to suggest objects that had special significance during lockdown, or photos that were typical of Norfolk people’s experience. Promotion on social media was a new way of working but critical to the project’s success. To ensure they were attuned to the national picture they also wanted to find out what was going on elsewhere in other museums, in the cultural sector and to keep abreast of what formal research was going on, particularly in health and welfare issues. A chance meeting with somebody at UEA put them in touch with people who had a good knowledge of projects that were going on. Finding those networks at the start of the project was important to make sure the work was not duplicating what others were doing and that information was shared. As a member of the team commented:

“*Our team could not let the change that is the impact of Covid-19 go unrecorded, so we straight away had to think about how are we going to obtain a full picture of the temporary and more enduring effects of the pandemic on the lives of the people of Norfolk*?... *Knowing who to go to in the areas you are less familiar with is key to the research we do.*”

**The Decolonisation Curatorial Working Group** was set up by staff in response to the Black Lives Matter movement to tackle racism within the collections by reviewing how items were acquired and the language used to describe objects. The group brings together 21 members from departments and museums across Norfolk. To research and develop appropriate methods staff are finding and drawing on information from regional organisations, other museums in the UK, and looking to the Museums Association to take a lead in the decolonisation debate. Whilst this is part of a bigger national and international agenda across museums, with no extra resources for this work, it was initiated and is being driven by the staff. As a member of the group stated:

“*We are all staff already working our hours and volunteering to do this work to take the museum forward…we don’t want to fall behind what other museums are doing, we want NMS to be at the forefront of decolonisation and to have an impact in that area.*”

Key strengths of this approach:

- The curatorial mind set fosters a culture of wanting to do research and making sure their work is backed up by research.
- A culture of research within the Service provides a level of autonomy that allows flexibility to take opportunities, but opportunities are restricted by timescales, budgets and especially by other commitments.
- Communication is essential, and a schedule to keep people communicating and to move projects forward.

### Case Study 4: The Environment Team - Embedded approaches to support research activities within the organisational structure and culture

The Environment Team are involved in various research activities and partnerships, including large externally funded projects, providing services and data to support external research, and engaging partners to conduct research related to Council activities. Examples of collaborative projects include: **Intereg European Regions and Intereg North Sea Region projects; EXPERIENCE** in which NCC is working with the University of Surrey and European partners to understand opportunities for sustainable ‘out of season’ tourism across Norfolk; **Staying Active and Independent Longer (SAIL)** which is about linking the value of the environment to clinical benefits for older people; and **Pushing Ahead** which is a collaboration between the Environment, Public Health, and Active Norfolk teams, and UEA to develop and evaluate interventions to….

The approach to research in the Environment team focuses on: developing mutually beneficial research relationships; integrating projects so that they are not considered in isolation, but in relation to the wider programme of work; and ensuring that research outputs can be used in a way that is beneficial to the work of the Council and the people of Norfolk. As members of the team stated:

*“We support all sorts of research by providing information and contacts with other experts, but if we are going to invest time and money into a project, we do it if we can see there would be a benefit for the people of Norfolk.”*

*“… You are constantly thinking about what next, so projects can be an inspiration but also produce evidence which can inform later projects.”*

Staff recognise that the team’s approach to research is innovative for a County Council. For example, bringing money in from external funding or commercial services has enabled innovation and engagement with novel projects that benefit the service and people of Norfolk. Members of the team described several benefits of research collaborations that they felt extended beyond the research outputs, as well as reflecting on some of the challenges, and approaches that have been key to the success of the work they do, and to expanding their partnerships:

- Working with universities brings access to academic expertise and advice; exposure to new ways of working that supports skills development and capacity-building; and credibility that helps to create momentum and buy in from other internal and external stakeholders. However, it can mean partners have slightly different objectives. Good communication is vital to ensure everyone is clear about objectives and expectations and how those will be managed. Researchers need to understand local government processes and limitations for collaboration to be effective.
- Project work can make long term planning difficult; a core team is needed to initiate and develop projects.
- Research relationships and activities are dependent on the project, how it is set up, and its purpose. Challenges can stem from differing collaboration arrangements, for example whether universities are a project partner or contracted for aspects of the research.
- Development and implementation of a platform for collaboration has made the process easier and audit proof.
- Internal engagement with other departments has built confidence around drawing other parts of the organisation into projects.
- Embedding staff with academic backgrounds has brought a skill set and connections for a research culture.
- Proactivity of staff in looking for opportunities to do research, to bring in external funding, and to develop partnerships has been important.
